# The expression and clinical significance of miR-132 in gastric cancer patients

**DOI:** 10.1186/1746-1596-9-57

**Published:** 2014-03-12

**Authors:** Xiaowen Liu, Hongmei Yu, Hong Cai, Yanong Wang

**Affiliations:** 1 Department of Gastric Cancer and Soft Tissue Sarcoma, Fudan University Shanghai Cancer Center, 270 Dong An Road, Shanghai 200032, People’s Republic of China; 2 Department of Oncology, Shanghai Medical College, Fudan University, Shanghai 200032, China

**Keywords:** Gastric cancer, Prognosis, Biological markers

## Abstract

**Background and objective:**

miR-132 plays a role in regulating neuronal morphology and cellular excitability. Little is known about the effects of miR-132 in cancer. The aim of this study is to evaluate the expression of miR-132 and its clinical significance in gastric cancer.

**Methods:**

Cancerous tissues and corresponding normal tissues from 79 patients with gastric cancer were examined for the expression of miR-132 using quantitative PCR and the association between miR-132 expression levels and clinicopathological factors and prognosis was analyzed.

**Results:**

In 79 clinical samples of gastric cancer patients, miR-132 expression levels in cancer tissues were significantly higher than those in the corresponding normal tissues (*P* = 0.001). Higher expression levels of miR-132 were associated with more frequent lymph node metastasis (*P* = 0.033), more lymphatic tumor emboli (*P* = 0.007), and more advanced stage (*P* = 0.016). Additionally, expression of miR-132 was an independent prognostic factor for overall survival (*P* = 0.020).

**Conclusion:**

miR-132 could serve as an efficient prognostic factor for gastric cancer patients.

**Virtual slides:**

The virtual slide(s) for this article can be found here: http://www.diagnosticpathology.diagnomx.eu/vs/8168577241196050

## Introduction

Although the incidence of gastric cancer has been substantially declining for several decades, it is still the fourth most common cancer and the second most frequent cause of cancer death [1,2]. A multiple of genes seem to contribute to the malignant biological behaviour of gastric cancer [3,4]. It is very important to identify the prognostic factors in order to maximize the therapeutic effect and to minimize the adverse effects in the treatment of cancer patients.

MicroRNAs are a class of endogenous small noncoding RNAs that function as gene regulators by regulating messenger RNA translation and degradation [5]. MicroRNAs play a crucial role in regulating the normal functions such as proliferation, differentiation, and apoptosis. Moreover, dysregulation of miRNAs contribute to the carcinogenesis and cancer progression [6,7]. As a member of miRNAs family, miR-132 plays an important role in inflammation, angiogenesis, and neural development [8-10]. Recently, some studies showed that the dysregulation of miR-132 was related to a variety of human tumors, such as non-small cell lung cancer, osteosarcoma, breast cancer, hepatocellular carcinoma, prostate cancer, and pancreatic cancer [11-16]. However, there are no reports about the expression and clinical significance of miR-132 in gastric cancer.

In this study, the miR-132 expression in gastric cancer patients was examined and the association between miR-132 and clinicopathological parameters was analyzed. Additionally, we evaluated the expression of some proteins involved in cell-cycle control (p21, p53), tissue proliferation and differentiation (c-myc). p21 and p53 proteins are the products of tumor-suppressor genes, which are activated by modulating cell proliferation via control of the G1 arrest checkpoint of the cell cycle [17,18]. c-myc protein is a type of transcription factor.

## Patients and methods

Human gastric cancer and corresponding normal tissues were obtained from 79 patients who underwent gastrectomy at the Department of Gastric Cancer and Soft Tissue Sarcomas, Fudan University Shanghai Cancer Center, between January 2007 and December 2009. Partial gastrectomy was performed in 36 patients, and total gastrectomy was performed in the other 43 patients. At least 4 cm resection margins from gross tumor were guaranteed for all patients. A total of 71 patients received adjuvant chemotherapy. After resection, the specimens were immediately frozen in liquid nitrogen and then stored in -80° refrigerator. Inclusion criteria for this study were adenocarcinoma and complete pathological data; Exclusion criteria were neoadjuvant therapy, peritoneal dissemination, and distant metastasis. Data were retrieved from their operative and pathological reports, and follow-up data were obtained by phone outpatient and clinical database. The written informed consent had been obtained from all the patients, and this study was approved by the Ethical Committee of Shanghai Cancer Center of Fudan University.

### RNA extraction and quantitative real-time PCR

The total RNA from tissue was extracted with TRIzol reagent (Invitrogen, Carlsbad, CA, USA) following the manufacturer’s instructions. Quantitative real-time PCR (qRT-PCR) assays were carried out to detect the endogenous expression of miR-132 according to the Applied Biosystems method. Briefly, cDNA was reverse transcribed from total RNA samples using a miRNA-specific stem-loop primer from the Taqman MicroRNA Assays and reagents from the TaqMan® MicroRNA Reverse Transcription Kit (ABI, Forest City, CA, USA). Then, PCR products were amplified from cDNA samples using the TaqMan MicroRNA Assays together with the TaqMan® Universal PCR Master Mix and the amount of PCR product were monitored using Applied Biosystems 7900 Sequence Detection System. U6 small nuclear RNA was used as an internal control. The relative amount of miR-132 was calculated by using 2(-Delta DeltaC(T)) method [19].

### Immunohistochemical staining

p21, p53, and c-myc were detected by immunohistochemical method. All primary antibodies and mouse monoclonal antibodies were purchased from Dako (Hamburg. Germany). Immunohistochemical staining was performed by the enhance labeled polymer system (ELPS) method. After overnight incubation at 4°C with anti-p21, p53, and c-myc antibody, sections were treated according to standard immunoperoxidase methods using a streptavidin biotin peroxidise complex kit (LSAB + Kit/HRP; Dako). The peroxidise reaction was then developed with diaminobenzidine (Dako). Negative control sections were subjected to the same procedure except that the first antibody was replaced by phosphate buffer saline (PBS) [20].

### Immunohistochemical staining scoring

All the slices were evaluated by two pathologists without knowledge of clinical outcome. The percentage of immunoreactive cells and staining intensities were evaluated in each sample. The percentage of immunoreactive cells was graded on a scale of 0 to 4: no staining is scored as 0, 1-10% of cells stained scored as 1, 11-50% as 2, 51-80% as 3, and 81-100% as 4. The staining intensities were graded from 0 to 3: 0 is defined as negative, 1 as weak, 2 as moderated, and 3 as strong, respectively. The raw data were converted to the immunohistochemical score (IHS) by multiplying the quantity and intensity scores. On the final analysis, the cases had a score of less than 1 were considered as negative, and ≥ 1 was regarded as positive [20].

### Statistical analysis

The patients’ features and clinicopathological characteristics were analyzed using the two-tailed Student’s *t*-test for continuous variable and χ^2^ test for categorical variable. Overall survival curves were calculated by Kaplan-Meier method, and the differences between survival curves were examined with long-rank test. The independent prognostic factors were examined by the multivariate survival analysis using Cox proportional hazards model. The accepted level of significance was *P* <0.05. Statistical analyses and graphics were performed using the SPSS 13.0 statistical package (SPSS, Inc., Chicago, IL).

## Results

### Clinicopathological characteristics

There were 62 males and 17 females (3.6:1) with a mean age of 58 years. There was 7 (8.9%) early gastric cancers and 72 (91.1%) advanced gastric cancers. According to histological type, well-differentiated tumors were observed in 3 (3.8%) patients, moderately-differentiated in 11 (13.9%) patients, and poorly-differentiated tumors in remaining 65 (82.3%) patients. Of 79 patients, 25 (31.6%) had tumors located in the upper third, 15 (19.0%) had tumors in the middle third, 36 (45.6%) had tumors in the lower third of the stomach, and 3 (3.8%) had tumors occupied two-thirds or more of stomach. Lymph node metastasis was observed in 57 patients, the metastasis rate was 72.2%. The distribution of pathological stage was as follows: 10 (12.6%) patients belonged to stage I, 24 (30.4%) II, and 45 (57%) III.

### The expression and correlation of miR-132, p21, p53, and c-myc

miR-132 levels in 79 cancerous (T) and corresponding normal tissues (N) were examined by qRT-PCR. Most tumor tissues (57/79, 72%) showed elevated levels of miR-132 compared to the corresponding normal tissues, with a median increase of 2.3-fold. miR-132 levels in cancerous tissues (mean ± SD, 0.35 ± 0.58) were significantly higher than those in the corresponding noncancerous tissues (mean ± SD, 0.15 ± 0.35, *P* < 0.05). The expressions of p21, p53, and c-myc were examined by immunohistochemical staining. p21 expression was positive in 64.6% of all gastric cancer tissues. p21 staining was observed in the nucleus of carcinoma cells. p53 expression was positive in 74.7% of all gastric cancer tissues. p53 staining was observed in the nucleus of carcinoma cells. c-myc expression was positive in 44.3% of all gastric cancer tissues. c-myc staining was observed in the cytoplasm of carcinoma cells. miR-132 expression was associated with p53 and c-myc. There was no correlation with p21. Additionally, p53expression was associated with c-myc (Table 1).

**Table 1:** Positive results of correlations among biological markers

**Biological markers**		**Correlation coefficients (r)**	**P value**
miR-132	P53	0.274	0.015
miR-132	c-myc	0.228	0.044
P53	c-myc	0.235	0.038

### Clinical significance of miR-132 in gastric cancer

**Figure 1:**
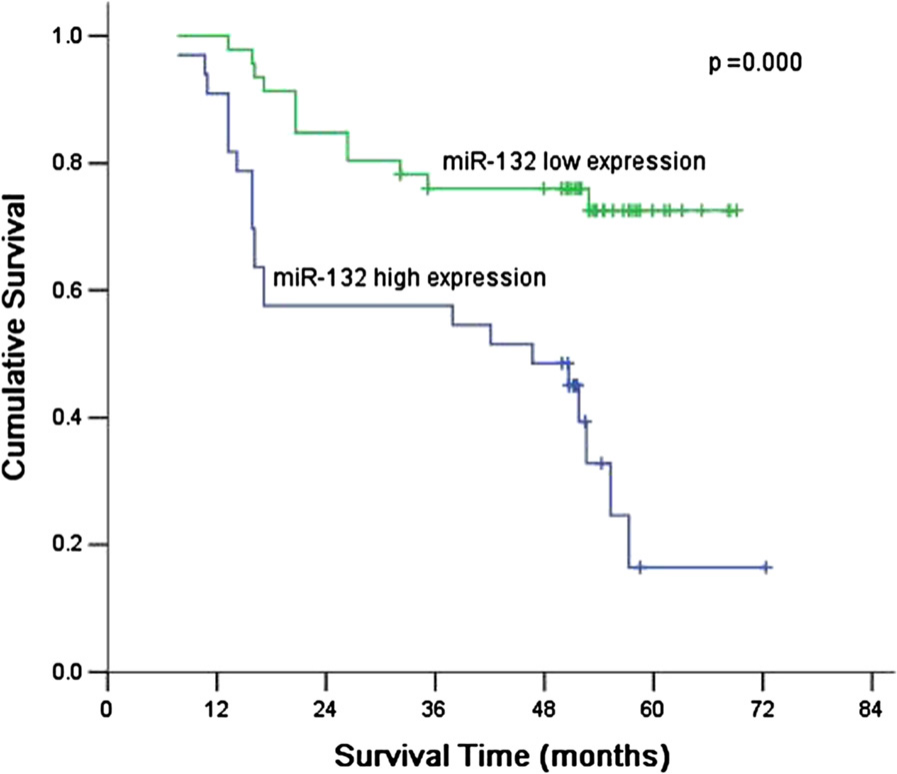
**Comparison of survival according to the expression of miR-132.** There were significant differences between miR-132 high expression and miR-132 low expression (*P* = 0.000).

79 gastric cancer patients were divided into two groups including miR-21 high-expression group (T/N >0.5, n = 33) and low-expression group (T/N <0.5, n = 46) according to the median T/N ration of miR-132 expression. The correlation between miR-132 expression and pathological parameters was analyzed. The results showed that patients with high miR-132 expression had more frequent lymph node metastasis (*P* = 0.033), more lymphatic tumor emboli (*P* = 0.007), and more advanced stage (*P* = 0.016). However, there were no significant differences about gender, age, histological type, tumor location, tumor size, nervous invasion, and T stage (Table 2). In the overall survival, the patients with the miR-132 high expression had a significantly poorer prognosis than those with miR-132 low expression (*P* = 0.000) (Figure 1). Univariate analysis of overall survival showed that the relative expression of miR-132, nervous invasion, lymphatic tumor emboli, and pathological stage were prognostic predictors. Multivariate survival analysis, including all significant prognostic factors mentioned in univariate analysis, was performed to determine the independent prognostic factors for gastric cancer. Multivariate analysis showed that the miR-132 expression level and pathological stage were independent prognostic factors (Table 3).

**Table 2:** miR-132 expression and clinicopathological factors

**Variables**	**High expression n = 33**	**Low expression n =46**	***P***
Gender			0.618
Male	25	37	
Female	8	9	
Age			0.556
<60	18	22	
≥60	15	24	
Histological grading			0.412
Poorly-differentiated	28	36	
Moderately-differentiated	5	7	
Well-differentiated	0	3	
Tumor location			0.467
Upper third	10	15	
Middle third	8	7	
Lower third	15	21	
Two-third or more	0	3	
Tumor size (cm)			0.862
<5	18	26	
≥5	15	20	
Nervous invasion			0.096
Yes	22	22	
No	11	24	
Lymphatic tumor emboli			0.005
Yes	26	22	
No	7	24	
T stage			0.070
T1,T2	6	17	
T3,T4	27	29	
Lymph node metastasis			0.033
Yes	28	29	
No	5	17	
Pathological stage			0.016
I	2	8	
II	6	18	
III	25	20	

**Table 3:** Multivariate analysis of patients by Cox model

**Variable**	**χ**^**2**^	***P *** **vale**	**RR**	**95% CI**
miR-132 expression	5.447	0.020	2.414	1.152-5.059
Nervous invasion	1.918	0.166	1.890	0.768-4.655
Lymphatic tumor emboli	0.173	0.677	0.838	0.366-1.923
Pathological stage	8.742	0.003	4.356	1.642-11.555

## Discussion

The identification of prognostic factors in gastric cancer was essential for predicting patients’ survival and determining optimal therapeutic strategies. The depth of invasion and lymph node metastasis were the most important prognostic factors in gastric cancer. As a result of the variability of prognosis within same pathological stage of gastric cancer, there have been a lot of researches for specific biological markers to identify patients with prognosis [21-28]. The biological markers such as microRNA, c-Met, L1CAM, endothelial lipase protein, variable copy number of mitochondrial DNA (mtDNA), TIMP3, hK6, and Ezrin have been studied extensively. As a novel biomarker, the potential of miRNAs in predicting prognosis is increasingly studied. miR-132 is a highly conserved miRNA transcribe from an intergenic region on human chromosome 17 by the transcription factor cAMP response element binding protein [29]. Most of the reports bout miR-132 regulation and biological functions emerged from the studies performed in the neuronal context. Additionally, miR-132 has also been described in a number of other fields, such as inflammation, cell transformation and tumourigenesis. miR-132 was shown to be up-regulated or down-regulated in different human cancers [16,30,31]. Some studies showed that miR-132 could promote cell proliferation [31]. However, there are still no reports on miR-132 in gastric cancer.

In this study, we investigated miR-132 expression in gastric cancer and determined if it could predict patient outcomes. We found that miR-132 expression was higher in cancerous tissues compared with corresponding normal tissues. The exact cause of the miR-132 high expression in gastric cancer is unknown. Park JK et al. reported [31] that the B2AR agonist terbutaline increases miR-132 expression. miR-132 was transcriptionally activated by CREB in neurons [32]. B2AR antagonists inhibited activation of CREB [33]. Therefore, it is possible that simulation of B2AR increase miR-132 expression following activation of CREB.

The results showed that high miR-132 expression in gastric cancer was significantly correlated with aggressive clinicopathological characteristics such as more frequent lymph node metastasis, more lymphatic tumor emboli, and more advanced stage. These results suggested that upregulation of miR-132 played an important role in gastric cancer progression. Interestingly, we found that miR-132 expression was associated with p53 and c-myc. Some previous studies showed that some microRNAs could mediate and regulate tumor suppression exerted by p53 or c-myc [34,35]. Jiang L et al. [34] found that has-miR-125a-3p could induce apoptosis via the p53 pathway. Zhang N, et al. [35] found that miR-150 could promote the proliferation of lung cancer cells by targeting p53. Wang B et al. [36] found that there was a double negative feedback loop between miR-122 and c-myc in hepatocellular cancer. Li X, et al. [37] found that c-MYC promoted the expression of mature miR-23a, miR24-2, and miR27a and subsequently promoted mammary carcinoma cell migration and invasion. However, the exact correlation between miR-132 and p53, c-myc is unclear and remains to be elucidated in future studies. We investigated the correlation of miR-132 expression with prognosis of gastric cancer patients, found that patients with high miR-132 expression had poorer overall survival that those with low miR-132 expression. More importantly, multivariate analysis revealed that high miR-132 expression was independent prognostic indicator. It was controversy whether miR-132 played same prognostic value in different kinds of tumors. Parker et al. reported [38] that high expression of miR-132 was associated with poor prognosis in patients with primary glioblastoma multiforme. However, Yang et al. [12] reported that osteosarcoma patients with low miR-132 expression had poorer overall and disease-free survival. It was possible that miR-132 played different functions in different tumors. Wang et al. [39] found that overexpression of miR-132 suppressed in vitro cell proliferation and in vivo tumor growth. Li et al. [13] showed that overexpression of miR-132 could inhibit the proliferation of breast cancer cell. Conversely, Park et al. [31] reported that over-expression of miR-132 increased the pancreatic cancer cell proliferation.

Taken together, our study indicated that miR-132 was upregulated in gastric cancer and might be an independent molecular biomarker for predicting the prognosis of gastric cancer. Meanwhile, as a result of small sample research, a large case research is needed to confirm the prognostic value of miR-132 in gastric cancer.

## Competing interests

The authors have declared that no competing interests exist.

## Authors’ contributions

Conceived and designed the experiments: XWL HMY HC YNW. Performed the experiments: XWL. Analyzed the data: XWL HMY. Contributed reagents/materials/analysis tools: HC YNW. Wrote the paper: XWL. All authors read and approved the final manuscript.
